# How to Find the Right RNA-Sensing CRISPR-Cas System for an *In Vitro* Application

**DOI:** 10.3390/bios12020053

**Published:** 2022-01-19

**Authors:** Escarlet Díaz-Galicia, Raik Grünberg, Stefan T. Arold

**Affiliations:** 1 Computational Bioscience Research Center (CBRC), Biological and Environmental Science and Engineering (BESE), King Abdullah University of Science and Technology (KAUST), Thuwal 23955-6900, Saudi Arabia; miriam.diazgalicia@kaust.edu.sa; 2 Centre de Biologie Structurale (CBS), INSERM, CNRS, Université de Montpellier, F-34090 Montpellier, France

**Keywords:** RNA sensing, CRISPR-Cas, spacer, *in vitro*, PAM, sensor design

## Abstract

CRISPR-Cas systems have a great and still largely untapped potential for *in vitro* applications, in particular, for RNA biosensing. However, there is currently no systematic guide on selecting the most appropriate RNA-targeting CRISPR-Cas system for a given application among thousands of potential candidates. We provide an overview of the currently described Cas effector systems and review existing Cas-based RNA detection methods. We then propose a set of systematic selection criteria for selecting CRISPR-Cas candidates for new applications. Using this approach, we identify four candidates for *in vitro* RNA.

## 1. Introduction

The natural CRISPR-Cas system comprises two main components. The CRISPR, acronym stands for “clustered regularly interspaced short palindromic repeats” and describes the first component—an array of short DNA fragments. These DNA fragments are snippets of bacteriophage genome sequences that bacteria and archaea retain from past infections. The RNA transcripts of these sequences are then recruited by the other component, the CRISPR-associated (Cas) proteins, where the transcripts serve as guide RNA (gRNA) to recognize subsequent infections by similar viruses. Upon forming the ribonucleoprotein (RNP) complex, Cas endonuclease activity specifically destroys the DNA or RNA of the invading virus [1,2,3,4,5,6,7,8,9,10]. This adaptive immune system of prokaryotes has now been repurposed into various programmable DNA editing tools [11,12,13,14].

Offering a large range of clinical, research, and diagnostic applications, CRISPR-Cas systems have rapidly become one of the most widely used molecular biology tools. While DNA editing remains the most prominent area of CRISPR applications, bioengineers have increasingly also turned their attention to more recently discovered Cas proteins capable of targeting and cleaving RNA instead of DNA [15,16]. Such RNA-targeting CRISPR systems are now being developed into impermanent, dose-dependent *in vivo* genetic therapies [17,18,19,20] or are used to identify RNA–protein interactions in living cells [21,22]. However, right after the discovery of the first RNA-targeting Cas ortholog (now named *Lsh*Cas13a) by Abudayyeh et al. [15], East-Seletsky et al. already demonstrated using these novel ribonucleases for detecting specific RNA sequences [16]. Consequently, *in vitro* RNA biosensing has become an important new application for CRISPR-Cas systems [23,24].

**Figure 1:**
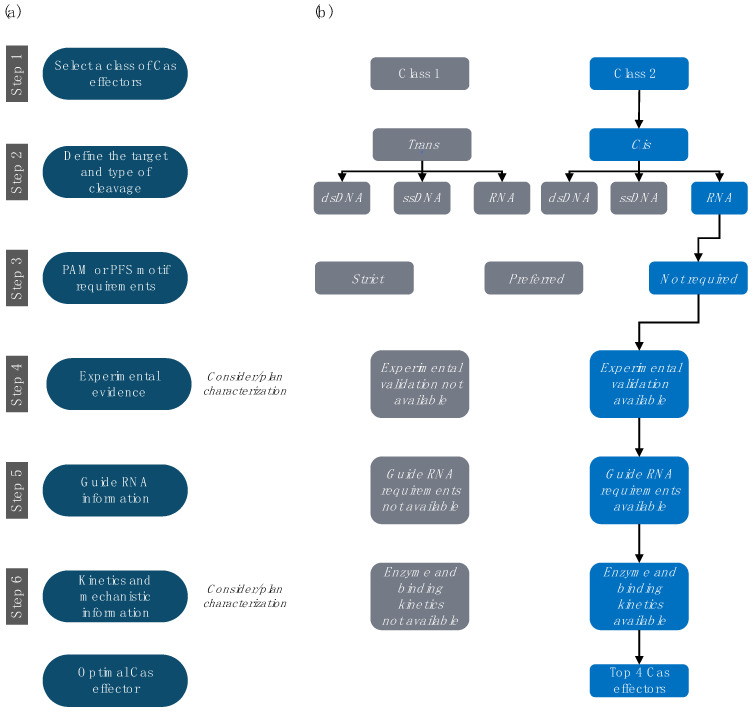
Selection criteria for RNA-targeting Cas. (**a**) General steps. (**b**) The choices leading to the four candidates described in greater detail are represented in blue.

The requirements substantially differ between cellular and *in vitro* applications, with the latter emphasizing biochemical factors, such as Cas expression yields, solubility and stability in buffers, and binding affinities or catalytic efficiency. As most CRISPR development still focuses on cellular applications and DNA-targeting Cas proteins, such data are not widely available for RNA-targeting CRISPR systems. Many *in vitro* Cas features that are critical for designing new applications, such as their mechanism of action, kinetics, and cleavage specificity, remain unknown or poorly documented. Therefore, selecting and characterizing the appropriate CRISPR-Cas ortholog for a given purpose becomes an essential but challenging task. Based on their evolution and architecture, Makarova et al. classified CRISPR-Cas systems into two classes, six types, and thirty-three subtypes [25]. We propose a step-by-step guide for identifying and prioritizing Cas effectors, specifically for *in vitro* applications (Figure 1). We concentrate on the underexplored RNA-targeting CRISPR systems and their use for *in vitro* RNA biosensors. Nevertheless, the selection criteria, tools, and methods we discuss equally apply to most *in vitro* uses of CRISPR-Cas systems.

## 2. Current CRISPR-Cas–Based RNA Detection Methods

**Figure 2:**
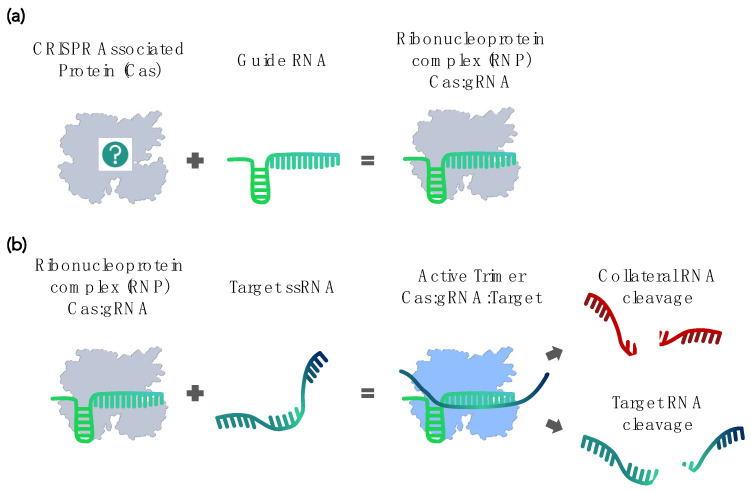
Schematic depiction of the RNA-targeting Cas system. (**a**) The Cas:gRNA complex comprises two parts: the Cas protein and guide RNA (gRNA). (**b**) The recognition and binding of target RNA activate the nuclease activity of the Cas:gRNA complex, resulting in the cleavage of target RNA. Some Cas orthologs, upon activation, also cleave nontarget, “collateral” RNA.

In 2016, Abudayyeh et al. demonstrated that the Cas ortholog *Lsh*Cas13a specifically targets mRNA in vivo but also displays an *in vitro* “collateral” cleavage activity, meaning that it nonspecifically cleaves nearby RNA in *trans* [15] (Figure 2). This nuclease activity in *trans* nevertheless strictly depends on the specific binding of the RNP complex to its actual RNA target [15]. It is fast and exhibits multiple turnovers, unlike the target cleavage, which is normally a single turnover [26]. That same year, East-Seletsky et al. used the *Lbu*Cas13a activity for the Cas-based RNA sensing of endogenous ß-actin mRNA from HeLa cell RNA extracts [16]. The collateral cleavage of single-stranded reporter RNA (ssRNA) molecules amplified target recognition events by up to 10,000-fold and translated them into a sensitive fluorescence readout. Many of the subsequently developed Cas-based nucleic acid detection methods still use variations of this same principle.

Currently, the most prominent CRISPR-based detection assays are SHERLOCK (specific high-sensitivity enzymatic reporter unlocking) and DETECTR (DNA endonuclease-targeted CRISPR trans reporter) [23,24]. Both use Cas orthologs (*Lwa*Cas13a and *Lb*Cas12a, respectively) to detect RNA (and DNA) with high specificity. Both rely on additional sample processing steps, such as (reverse) transcription reactions (i.e., RT-RPA, T7 *in vitro* transcription) and enzymatic DNA amplification to reach attomolar (aM) sensitivity. For SHERLOCK, the target RNA is reverse-transcribed before amplification and subsequently transcribed back into RNA for Cas binding. The Cas ortholog used by the DETECTR system can only detect DNA. Therefore, the target RNA is first reverse-transcribed and then amplified as DNA, which is recognized by the Cas [23,24]. Both systems collaterally cleave a reporter molecule upon binding the Cas effector to its target.

The SHERLOCK, DETECTR, and similar Cas-based nucleic acid detection systems employ various approaches for converting reporter cleavage into a readout. The most common are fluorescence or colorimetry in solution or the capture of dyes on lateral flow strips. Fazouni et al. (2020) adapted *Lbu*Cas13a to the detection of the severe acute respiratory syndrome coronavirus 2 (SARS-Cov2) and used the camera of a mobile phone to sense and quantify RNA cleavage [27]. Another system, iSCAN (*in vitro* Specific CRISPR-based Assay for Nucleic acids detection), combines RT-LAMP (reverse transcription loop-mediated isothermal amplification) with *Lb*Cas12a, *Aac*Cas12b, or *Aap*Cas12b to detect SARS-Cov2 through a colorimetric readout on lateral flow cells [28]. SHERLOCKv2 multiplexes different Cas orthologs (*Psm*Cas13b, *As*Cas12a, and *Cca*Cas13b). Each Cas ortholog has a particular nucleotide-cleavage preference that facilitates the orthogonal detection of several targets in a one-pot reaction [29]. Similarly, HOLMES (one-hour low-cost multipurpose highly efficient system) and HOLMESv2 use *Lb*Cas12a and *Aac*Cas12b orthologs, respectively, to detect DNA and RNA with aM sensitivity [30,31].

Cas effectors have also been actively developed into more field-deployable platforms. For example, a CRISPR/Cas13a powered portable electrochemiluminescence chip (called PECL-CRISPR) detects miRNA by combining the sensitivity of *Lbu*Cas13a with the low background and large dynamic range of chemiluminescence and electrochemical detection. This system reports the detection of 1 femtomolar (fM) of miR-17 on a paper-based bipolar electrode [32]. A strategy inspired by digital PCR, SATORI (for CRISPR-based amplification-free digital RNA detection) combines *Lwa*Cas13a with the parallel observation of multiple reactions in femtoliter microchamber arrays and could detect 10 fM of ssRNA in less than 5 min [33].

In conclusion, different studies have combined various Cas orthologs with different signal amplification and readout strategies. In many cases, the rapid collateral cleavage activity of Cas13 systems is used to enzymatically amplify the response by unquenching the fluorescent signal from a reporter molecule (SHERLOCK, DETECTR [23,24]). Other methods use external reporting systems, such as electrochemical transistors, lateral flow paper strips, colorimetry, or electrochemiluminescence [27,28,32]. The requirements of the desired output system will be an important factor in the selection of a Cas ortholog. However, the reasons for selecting one ortholog over another with similar features are not always clear.

## 3. Computational Tools for CRISPR Identification

Computational protein prediction from sequences combined with experimental work has resulted in a vast and continuously increasing list of CRISPR-Cas systems. Many online tools help identifying new CRISPRs, spacers, and Cas proteins using sequence analysis. Several Cas system databases have been compiled. For example, Tang et al. constructed the Cas Protein Data Bank that catalogs Cas proteins from bacteria and archaea [34]. CRISPRminer attempts to describe the full biological picture of CRISPR genes, classification, spacers, and targets in several thousand bacterial and archaeal species [35]. CRISPRCasFinder [36] identifies CRISPR arrays, DRs, spacer sequences, and Cas genes along with their type and subtype in user-provided sequences. The associated CRISPRCasdb database catalogs this information for all known bacterial and archaeal genomes [37]. In order to facilitate the selection of RNA-targeting Cas effectors from the large number of theoretical candidates, we propose a step-by-step approach that is described in the following.

## 4. Step-by-Step Selection and Design of RNA-Targeting CRISPR-Cas Systems

### 4.1. Step 1. Select the Preferred Class of Cas Effectors

There are two main classes of Cas effectors (Table 1). Class 1 generally relies on multiple Cas subunit proteins to degrade foreign genetic elements (currently with only one exception [38]). Class 2 combines gRNA processing, target recognition, and cleavage activities into a single multidomain protein. Reducing the number of parts also reduces the complexity of engineering. Moreover, the published research on Cas applications generally utilizes Class 2 effectors. Therefore, we do not consider Class 1 CRISPR-Cas systems in our selection. Genomic and metagenomic data analyses identify more than 175,000 Class 2 Cas family members [39]. This number is constantly increasing due to computational predictions, protein screens, and new sequencing data analyses.

### 4.2. Step 2. Select the Preferred Target and Type of Cleavage

The three main targets for Cas endonucleases are double-stranded DNA (dsDNA), single-stranded DNA (ssDNA), and RNA, and we focus on the latter. The first identified RNA-targeting CRISPR-Cas systems belonged to the Class 1 Type III-B (*Pyrococcus furiosus*, Cmr) [40]. However, we excluded Class 1 enzymes in Step 1 because of their multiple-effector architectures. Despite possessing a single-effector nuclease, we also excluded the highly diverse Class 2 Type V effectors because their main cleavage target is dsDNA or ssDNA (i.e., Cas12 and Cas14), although some cleave “bystander” ssRNA in a *trans*/collateral way (i.e., Cas12). Only Cas effectors belonging to Class 2 Types II and VI are likely to recognize and cleave ssRNA and, therefore, were further considered.

While we did not specifically consider it in our example, collateral cleavage activity may often become an additional selection criterion. Efficient collateral RNA cleavage is a hallmark of Type VI (Cas13) Cas proteins but, as mentioned, can also be found in some Type V Cas orthologs [41]. Evidently, cleavage of “bystander” RNA may not be desirable for all applications but is often used for signal amplification and readout. Interestingly, certain Cas orthologs show a nucleotide or sequence bias also for *trans*-cleaved substrates. Such a property may add a secondary degree of programmability, which could improve a device’s fidelity [29].

### 4.3. Step 3. Investigate PAM or PFS Motif Requirements

Protospacer adjacent motifs (PAMs) and protospacer flanking sites (PFSs) are both short sequences (~1–6 nt) immediately following the target sequence (DNA or RNA, respectively). Both PAMs and PFSs are “hard-wired” into the enzyme’s specificity, and, while they need to be present in the target, they are not encoded in the gRNA or its encoding CRISPR array. Thus, they protect the host CRISPR locus from cleavage by the host’s own Cas systems. These elements are indispensable for target recognition in many Cas orthologs (i.e., *Sp*Cas9, *Aac*Cas12, and *Bth*Cas12). Consequently, on the one hand, the PAM or PFS sequence must be present in the target and cannot be reprogrammed. This constraint reduces the number of possible target sites on a given sequence. On the other hand, PAM or PFS often improve the target specificity of the system. Some Cas orthologs can target ssDNA independently of a PAM but require a PAM for dsDNA targets [42]. For ssDNA and RNA targeting, attempts have been made to circumvent the PAM constraint using protein engineering [43].

The PFS sequences are localized in the 3′ of target ssRNA and affect the efficacy of some obligatory ssRNA-targeting Cas proteins of Type VI but are not a strict requirement for RNA targeting in mammalian cells [17,44]. However, many RNA-targeting Cas effectors, such as *Lwa*Cas13a (Type VI), *Es*Cas13d (Type VI), and *Sau*Cas9 (Type II), do not require any PFS sequences for their *in vitro* activity [45]. We opted to prioritize the Cas proteins from Type II and VI without any requirement for PAM/PFS (respectively) to maximize our flexibility in selecting targets. However, dsDNA-specific Cas systems with PAM requirements may be “tricked” into the recognition and cleavage of ssDNA or RNA by the design of so-called PAMers [26,46,47]. PAMers are synthetic oligonucleotides that hybridize with a single-stranded target RNA (or DNA) and create a short double-stranded segment, including the enzyme’s PAM. There is also emerging evidence for the successful use of PAMers for RNA-targeting Cas proteins (i.e., *Sa*Cas9, *Spy*Cas9, *Nme*Cas9, and others.) [46,48].

### 4.4. Step 4. Prioritize Effectors with Experimental Characterization

Even after limiting the search to RNA-targeting Class 2 Type II and VI without PSF requirements, the number of available orthologs is still very large. For instance, CRISPRminer reports about 4300 entries of Cas proteins from Class 2 Types II and VI [35]. Given that the success rate in bioengineering is dramatically increased if a certain biological part has already been characterized experimentally, we prioritize Cas effectors for which such data are available.

As we focus on *in vitro* applications, the primary criterion is the availability of experimental *in vitro* evidence for RNA target cleavage or binding. A widely used method is the electrophoretic mobility shift assay (EMSA) [57], in which the binding of the Cas complex delays the migration of the target RNA/DNA through a gel. Alternative methods include filter binding assays [26,58] or biochemical cleavage assays [26,30,42]. In addition, fluorescence-based assays can provide proof of protein functionality, the mechanism of action, and target specificity [58,59]. Quantitative data on binding affinity are scarce but much preferred over qualitative results. These are often derived from densitometry quantification of EMSA bands (i.e., for *Sau*Cas9 [26]) or, more accurately, by biophysical methods, such as isothermal titration calorimetry, microscale thermophoresis, or surface plasmon resonance [60].

The documentation of protein expression and purification protocols is also important for *in vitro* applications. Studies rarely report their quantitative protein yield. Where data are available, we used them to prioritize Cas effectors that required a smaller number of purification steps and for which high solubility and detailed purification protocols are reported (i.e., *Lwa*Cas13a) [42]. We excluded Cas effectors without purification reports or those lacking results in controlled environments. In this case, this step eliminated *Pgu*Cas13b, *Psp*Cas13b, and *Rfx*Cas13d. We also excluded some Cas orthologs, such as CasRx, which perform well in cell cultures (mammalian, insect, plant, or bacterial) but whose activity has not been characterized *in vitro*.

### 4.5. Step 5. Investigate gRNA Information

In native CRISPR-Cas9 systems, the gRNA consists of two base-paired RNA molecules: (i) a CRISPR RNA (crRNA) that encodes a sequence (spacer) that is complementary to the target and (ii) a trans-activating CRISPR RNA (tracrRNA) that mediates the interaction of the Cas effector protein with the crRNA [61]. A major initial step for enabling the use of CRISPR-Cas as a programmable DNA editing tool was the design of a single gRNA (sgRNA) that directed the Cas protein (and its associated enzymatic activity) to a chosen DNA target as defined by the complementary gRNA sequence [12]. Some Class 2 effectors, such as Cas13, naturally use only a single crRNA molecule. This natural gRNA contains a direct repeat (DR) stem-loop that mediates the interaction with the Cas protein and a spacer sequence that determines the target selectivity [62]. The terms gRNA, sgRNA, crRNA, and spacer are sometimes used interchangeably in the literature.

The exact gRNA design rules for a given Cas ortholog must be known before adapting the system to any application. At a minimum, this includes the spacer length requirements and the sequence and structure details of the DR. More detailed information on spacer specificity is preferable. For example, spacers of Cas9 orthologs usually feature a seed region (usually 8–10 nt at the 3′ end of the spacer) with low or no mismatch tolerance [12]. This seed region participates in the first target interrogation step [12,63]. For generally well-characterized Cas systems, we find quantitative data on the mismatch tolerance or sensitivity across the full spacer sequence (a particularly good example is *Lbu*Cas13a [58]). Target recognition may depend on divalent ions, such as Mg^2+^ and Mn^2+^, which is an important consideration for the experimental design (i.e., *Es*Cas13a [64]). Even if the gRNA architecture is well understood, the selection of the actual target-specific spacer sequence may still substantially affect the functioning of the detection system. Several bioinformatics tools aim to help select spacer sequences for Cas9 systems targeting DNA [65]. Tools for non-Cas9 systems have also been developed and often consider the secondary structure of both the gRNA and target RNA [66,67,68]. For example, Wessels et al. created an online tool that predicts Cas13d gRNAs for all protein-coding transcripts in the human genome (cas13design.nygenome.org) [62]. If not already considered by the primary tool, secondary structure predictors, such as RNAfold [69] and RNAxs [70], may be employed as a secondary filter. Nevertheless, a strong consensus exists in the literature that at least two to five spacer sequences must be tested experimentally to determine the most efficient guide for a particular target and working conditions (with other variables including the buffer, temperature, or target binding kinetics) [27].

In the example analysis, we prioritized Cas effectors with detailed gRNA characterization data and reduced mismatch tolerance within and outside the seed region. Examples of such systems are *Lbu*Cas13a and *Lwa*Cas13a [15,58]. Coincidentally, both of these Cas orthologs can process or maturate their crRNA from a tandem array. Depending on the application, this activity could offer an opportunity for efficient multiplexing. However, this feature was not considered in the prioritization.

### 4.6. Step 6. Review Available Kinetics and Mechanistic Information

The Cas proteins have markedly different sizes (currently ranging from ~800 to 1700 aa) [71,72] and domain compositions (i.e., RucV versus HEPN-nuclease motifs). The smaller size of single-effector proteins facilitates their transfection into cells, both directly using protein transfection methods or through viral vectors (i.e., Cas13bt, d, e, and f [71,72,73]). Conversely, larger Cas proteins may have additional (beneficial) functions, such as the RNase modules that process pre-crRNA. Type II and VI effectors usually adopt a bi-lobed architecture (the recognition (REC) lobe and nuclease (NUC) lobe) typically connected by an arginine-rich bridge helix and linker loop [26].

For most Cas effectors, the mechanism by which the gRNA interacts with the Cas protein (duplex complex) and how this binary complex interacts with the target (triple complex) is still not completely understood. Structural analyses have indicated that Cas catalytic activity and specificity often depend on large conformational changes when moving from duplex to triple complexes [41,74]. In general, three-dimensional (3D) structural information is an important tool for informed engineering and testifies to the successful recombinant expression and purification of a protein. Therefore, we prioritized the Cas effectors for which structures were published. Additionally, we kept *Lwa*Cas13a, where a detailed record of biochemical characterization compensates for the lack of a structure.

Where quantitative comparisons were available, we preferred Cas orthologs with the higher catalytic activity (e.g., *Lwa*Cas13a rather than *Lsh*Cas13a) [44]. Although relevant, we found that the affinity of the interaction between the Cas effector and its gRNA is generally lacking. However, apparent dissociation constants (K_d_s) are sometimes reported for the binding of the Cas–gRNA complex to its targets [26]. The availability of these data was the final criterion that promoted *Sau*Cas9 and *Lwa*Cas13a to the list of top notable candidates. Moreover, specific applications or experiments (e.g., control reactions) may require catalytically dead Cas proteins. We prioritized Cas effectors for which (i) target binding had been reported with both active and catalytically dead versions and (ii) the full description and (if possible) structural analysis of these inactivating mutations were available [17].

## 5. Detailed Profiles of Four Cas Effectors for *In Vitro* RNA Detection

**Figure 3:**
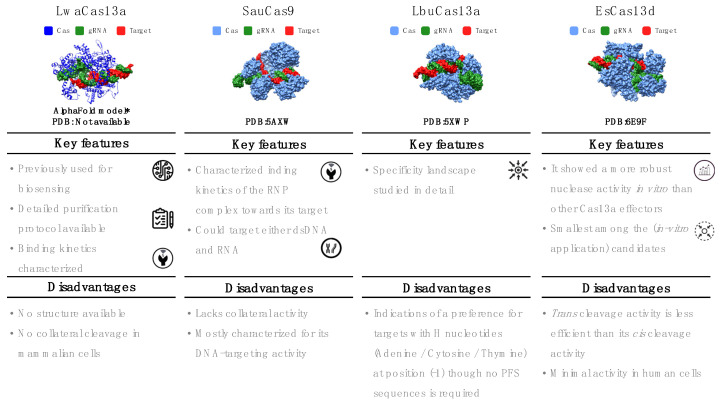
Key features of selected Cas effectors.

The six-step decision workflow led us to select four Cas systems that we consider top candidates for developing *in vitro* RNA detection systems (Figure 3). As we highlighted above, some of the selection criteria were strict constraints, such as the restriction to Class 2 enzymes or the availability of gRNA design rules. Other criteria resulted from the current scarcity of experimental data and sometimes had to be weighed against each other. These latter criteria can be reassessed when more experimental data are reported.

### 5.1. Leptotrichia wadei (LwaCas13a)

This single-effector nuclease is mostly known for its use in the SHERLOCK system. Its 3D structure has not yet been determined, but *Lwa*Cas13a has been extensively characterized for several *in vitro* and in vivo applications. *Lwa*Cas13a is an RNA-only targeting nuclease with *cis* and collateral/*trans* cleavage *in vitro* and in bacterial cells [16,42,44]. However, in mammalian cells, no collateral cleavage has been observed [44]. *Lwa*Cas13a does not require a PFS sequence. However, like other Cas13 orthologs, Cas:gRNA binding to the target can be hindered by strong secondary structure motifs in the target [16,44]. The catalytically inactive *Lwa*Cas13a version maintains binding to the target [44]. Its natural spacer length is 29–30 nt. Although *Lwa*Cas13a retains its cleavage activity with spacer lengths as short as 20 nt [44], most studies have used 28 nt spacers. The SHERLOCK assay successfully detects 20 fM of non-amplified ssRNA even after lyophilization and rehydration of *Lwa*Cas13a. When combined with nucleic acid amplification, the sensitivity of SHERLOCK reaches 2 aM [24]). *Lwa*Cas13a is incapable of cutting sequences with two or more mismatches. Consequently, spacers with one deliberate mismatch in the guide sequence allow the detection of targets with perfect sequence specificity. Single-nucleotide polymorphisms or closely related pathogen strains can be distinguished using this method [29,42]. Besides nucleic acid detection in clinical samples [24,29,75], *Lwa*Cas13a has been used to detect plant and food pathogens [76,77].

### 5.2. Staphylococcus aureus (SauCas9)

The single-effector Cas *Sau*Cas9 belongs to Class 2 Type II. With a molecular weight of 124 kDa, *Sau*Cas9 is markedly shorter than other Cas9 effectors (typically ~160 kDa) [78,79]. It possesses two nuclease domains (RuvC and HNH) and can cleave both dsDNA and RNA, but not simultaneously [26,78]. The RNA-cleavage activity has a strong preference for nonstructured/ssRNA targets. Unlike other RNA-targeting effectors, *Sau*Cas9 does not have collateral ribonuclease activity. It requires a relatively long PAM sequence (5′-NNGRRT-3′) for DNA targeting, but neither PAM nor PFS are required for RNA cleavage [26]. The literature on DNA targeting is extensive [78,80,81,82,83] and includes crystal structures with DNA targets (Protein Data Bank (PDB): 5AXW and 5CZZ) [78]. RNA targeting is less well studied but has been confirmed through *in vitro* cleavage assays, filter binding, and EMSA experiments [26]. Kinetic data suggest that *Sau*Cas9 is a multiple-turnover enzyme for DNA targets but a single-turnover enzyme for RNA targets [26,82]. The dissociation constant for the binding of *Sau*Cas9:gRNA to target RNA is about 1.8 nM [26,82]. SauCas9 requires divalent ions for cleavage. Its optimal spacer length is 23 nt, and the gRNA sequence is available [26,82]. A catalytically dead version (d*Sau*Cas9) and a split-protein version for DNA or RNA binding and recognition inside cells have been described [78].

### 5.3. Leptotrichia buccalis (LbuCas13a)

This Cas system is a single-effector protein of about 140 kDa. Similar to other effectors in Class 2 Type VI, it targets ssRNA and possesses both *cis* and *trans* cleavage activity in the presence of Mg^2+^ [16]. *Lbu*Cas13a can process its pre-crRNA, but the processing of the crRNA is not required to activate the effector protein [16,41,84,85]. Its specificity landscape has been studied in detail, concluding that this protein can distinguish between fully complementary and mismatched RNA transcripts (even those containing only a single mismatched nucleotide) before activating the RNP complex [58]. *Lbu*Cas13a is a sensitive RNA detector with observable reporter cleavage in the presence of only 10 fM of the activator target. Its structure has been determined (PDB:5XWP and 5XWY [84]), revealing conformational changes both after binding to the gRNA and after forming the triple complex with the target [84]. The target RNA must be at least 20 nt in length to activate the Cas effector [84]. The cleavage activity of *Lbu*Cas13a has been characterized in cell-free experiments through EMSA, filter binding, fluorescence assays, and fluorescence polarization [16,58]. *Lbu*Cas13a does not require a PFS sequence [58]. However, there are indications of a preference for targets with H nucleotides (adenine/cytosine/thymine) at position (-1) [58]. The sequence of the constant region of its gRNA has been published (Table 2) [16,41,58,74,84,85]. The seed region (gRNA nucleotides 9–14 nt) required for target binding is mismatch hypersensitive, whereas mismatches in the HEPN-nuclease switch region (gRNA nucleotides 5–8) can result in an inactive nuclease that remains tightly bound to the target [58].

### 5.4. Eubacterium siraeum (EsCas13d)

Derived from a gut-resident bacterium, *Es*Cas13d (~105 kDa) was among the two first characterized single-effector Cas of Class 2, Type VI, Subtype d [71,74]. *Es*Cas13d is an ssRNA-only nuclease [71]. It exhibits *cis* and *trans* cleavage activity, with *trans* less efficient than *cis* [71]. Structures for both apoprotein and binary (Cas:gRNA) or ternary (Cas:gRNA: target) complexes have been determined (Electron Microscopy Data Bank (EMDB): 9015, 9013, and 9014 and PDB: 6E9E and 6E9F [74]). Although *Es*Cas13d exhibited minimal activity in human cells, *in vitro* experiments demonstrated more robust nuclease activity than Cas13a effectors [71]. *Es*Cas13d does not require a PFS sequence. However, Mg^2+^ is necessary for target cleavage and can improve the efficiency of crRNA maturation. Additionally, evidence exists that two hydrated Mg^2+^ ions stabilize the conformation of the DR region [64]. *Es*Cas13d can process its crRNA, a single-molecule guide comprising a DR region (of about 30 nt located, unlike others, at the 5′ end) and a spacer region [74].

Somewhat contradictory reports describe a solvent-exposed seed region, either in position 1–16 nt or 5–21 nt of the spacer, intolerant to two consecutive mismatches [62,74]. *Es*Cas13d is considered a highly specific effector because its activation depends not only on binding the correct target sequence, but is in addition gated by a very profound conformational change [66]. Yan et al. found that an optimal cleavage activity is achieved with a 21–30 nt spacer, and the most commonly reported spacers are 23 nt long [86]. As other Cas proteins, *Es*Cas13d prefers a low secondary structure content in the targeted RNA [66,86]. It has been experimentally characterized using biochemical cleavage assays and filter binding assays for which crRNA and target sequences were reported [66,74]. Both catalytically active and inactive Cas variants expressed well in *Escherichia coli* [71].

### 5.5. Other Notable Cas Candidates

Throughout the literature review, we encountered additional Cas effectors that, although they do not comply with all suggested criteria, possess features that may benefit particular applications. For example, *Ruminococcus flavefaciens* XPD3002 (CasRx or *Rfx*Cas13d) has been recognized and intensely studied for its high RNA knockdown efficacy with minimal off-target activity in human cells [62]. Another example is *Lb*Cas12a, a Cas12 ortholog used in the well-established diagnostic tool DETECTR, which is a strict DNA endonuclease (but was combined with reverse transcription for RNA detection [23,87]). Finally, while editing this review, Li et al. (2021) reported the cryo-EM structure of Cas12g. This subtype specifically recognizes and requires ssRNA for its activation but exhibits both collateral DNA and RNA cleaving capabilities [88].

## 6. Conclusions

We summarized recent technologies for RNA detection with Cas proteins and provided a detailed description of the most important features to search for when selecting a Cas effector. Computational screens have already identified thousands of Cas orthologs targeting both DNA and RNA, yet we have barely started exploring the rich CRISPR toolbox that nature offers. Experimental characterization of Cas systems has largely focused on cell applications, whereas *in vitro* characterization data, if available, are often buried in the supplementary information. However, the informed design of *in vitro* biosensors critically depends on this biochemical characterization, which often reveals important deviations from the efficacy and selectivity observed in cells [71]. Bioengineering studies must describe how and why a particular Cas ortholog was selected. While it may seem safer to base a new design on a Cas system that many others have already employed, the initial choice of this system may have been arbitrary. We urge the CRISPR bioengineering community to document such design choices and help expand the field of well-characterized candidates.

## Figures and Tables

**Table 1:** Features of CRISPR-Cas systems by type.

Class	Features	Type	Features	Key Effectors	Target	Ref.
1	Effector, adaptation, and accessory functions distributed over multiple proteins	I	Assembly of multiple Cas proteins into the signature CRISPR-associated complex for antiviral defense (Cascade)	Cas1, Cas2, Cas4, Cas5, Cas6, Cas3, Cas8	dsDNA	[25,49,50]
		III	Signature multimeric complex known as Csm/Cmr Some effectors in this type use spacers produced by Type I systems	Cas1, Cas2, Cas5, Cas6, Cas7, Cas10	dsDNA, RNA	[51,52]
		IV	Often lacks adaptation module genes (Cas1 and Cas2) Involved in competition between plasmids in bacteria	Cas5, Cas7, Csf1	dsDNA	[52,53]
2	Single protein with multiple domains combines crRNA-binding, catalytic activity, and pre-crRNA processing	II	Mainly DNA binding Contains two metal-dependent nuclease domains (HNH and RuvC) Requires PAM Relies on RNase III to process its crRNA	Cas9	dsDNA, RNA	[12,25,54,55]
		V	Requires PAM Reduced off-target activity compared to Cas9 Collateral RNA or ssDNA cleavage in some subtypes	Cas12, Cas14	dsDNA, ssDNA, RNA	[25,56]
		VI	Two HEPN nuclease domains collateral RNA cleavage Some orthologs inactive in mammalian cells No PFS dependency in some subtypes Processes its own crRNA	Cas13	RNA	[15,25,41,54]

**Table 2:** Feature summary for the four selected Cas effector candidates.

Identifier	Source Organism	Features	gRNA
***Lwa*** **Cas13a**	*Leptotrichia wadeii*	**Size**: 1389 aa **Structure**: not available **Nuclease domain**: HEPN **dCas mutations**: D403G, R474A, and R1046A [44] **PFS**: not required **Optimal spacer length**: 20–28 nt **Specificity**: collateral cleavage *in vitro* but not in mammalian cells **Turnover kinetics**: not available **Others**: used for SHERLOCK diagnostics	5′-GATTTAGACTACCCCAAAAACGAAGGGGACTAAAAC-*SPACER*
***Sau*** **Cas9**	*Staphylococcus aureus*	**Size**: 1053 aa **Structure**: 5AXW **Nuclease domain**: RuvC and HNH **dCas mutations**: D10A (RuvC), N580A (HNH) [26] **PAM/PFS**: PFS not required for ssRNA targeting; PAM required for dsDNA targeting (5’NNGRRT) **Optimal spacer length**: 23 nt **Specificity**: **High** DNA target specificity due to long PAM; mismatch tolerance characterization available for its RNA-targeting role; no collateral activity **Turnover kinetics**: DNA (multiple), RNA (single) **Others**: target secondary structure affects affinity	5′-*SPACER*- GTTTTAGTACTCTGGAAACAGAATCTACTAAAACAAGGCAAAATGCCGTGTTTATCTCGTCAACTTGTTGGCGAGATTT
***Lbu*** **Cas13a**	*Leptotrichia buccalis*	**Size**: 1159 aa **Structure**: 5XWP **Nuclease domain**: HEPN **dCas mutations**: R472A, H477A, R1048A and H1053A [84] **PFS**: not required **Optimal spacer length**: 20–24 nt **Activity**: collateral activity with high turnover (10^4^ turnovers per target RNA recognized) [41] **Turnover kinetics**: not available for target cleavage but multiple turnover for collateral cleavage	5′- GGCCACCCCAAAAATGAAGGGGACTAAAACA-*SPACER*
***Es*** **Cas13d**	*Eubacterium siraeum*	**Size**: 954 aa **Structure**: 6E9F **Nuclease domain**: HEPN **dCas mutations**: R295A, H300A, R849A and H854A [71] **PFS**: not required **Optimal spacer length**: 20–30 nt **Specificity**: collateral activity **Turnover kinetics**: not available **Others**: robust expression in *E. coli*; limited activity in mammalian cells [71]	5′ AACTACACCCGTGCAAAAATGCAGGGGTCTAAAAC-*SPACER*
